# Genetic Diversity and Drug Resistance Mutations in HIV-1 from Untreated Patients in Niamey, Niger

**DOI:** 10.5402/2011/797463

**Published:** 2011-11-03

**Authors:** Saïdou Mamadou, Yahayé Hanki, Amadou Roufaï Ali Maazou, Balki Aoula, Sanata Diallo

**Affiliations:** ^1^Laboratory of Bacteriology-Virology, Faculty of Health Sciences, Abdou Moumouni University, P.O. Box 237, Niamey, Niger; ^2^National Reference Laboratory for STI/HIV/TB, P.O. Box 10 146, Niamey, Niger; ^3^Centre de Traitement Ambulatoire (CTA) of Niamey, P.O. Box 11 236, Niamey, Niger; ^4^Solidarité Thérapeutique et Initiative Contre le Sida (SOLTHIS), P.O. Box 10 393, Niamey, Niger

## Abstract

The objective of the study was to estimate the prevalence of transmitted resistance to antiretroviral of HIV-1 circulating in Niger. We collected plasmas from 96 drug-naive patients followed up in the main HIV/AIDS Care Center of Niamey, the capital city of Niger. After RNA extraction and retrotranscription to proviral DNA, nested PCR was performed to amplify PR (codons 1–99) and RT (codons 1–240) fragments for sequencing. Sequences were analysed for phylogeny, then for resistance-associated mutations according to IAS-USA and Stanford's lists of mutations. We characterized six HIV-1 genetic variants: CRF02-AG (56.3%), CRF30_0206 (15.6%), subtype G (15.6%), CRF06_cpx (9.4%), CRF11_cpx (2.1%), and CRF01_AE (1%). About 8.3% of HIV strains had at least 1 resistance mutation: 4 strains with at least 1 mutation to NRTI, 5 for NNRTI, and 1 for PI, respectiveley 4.2%, 5.2%, and 1.0%. These preliminary results gave enough information for the need of instauring HIV drug resistance national surveillance.

## 1. Introduction

HIV-1 strains belong to four groups of genetic variants (M, N, O, P), which may represent four separate introductions of simian immunodeficiency viruses to humans. Into group M, mutations caused by retrotranscription errors generated nine subtypes (A, B, C, D, F, G, H, J, and K) of HIV-1 [1]. Genetic recombination events during multiple infections provided various mosaic viruses: 49 circulating recombinant forms (CRFs) to date (http://www.hiv.lanl.gov/content/sequence/HIV/CRFs/CRFs.html) and many unique recombinants. Almost all these HIV-1 genetic variants co-circulate in Africa. In Niger, a sub-Saharan country with HIV prevalence under 1% in general population, none-B subtypes, and CRFs cocirculate, with large predominance of CRF02_AG and CRF06_cpx [2, 3]. 

The National Programme for Antiretroviral (ARV) Therapy Access started in this country in November 2004 more than six thousand people were treated, at the end of 2010. First-line association was stavudine/lamivudine/nevirapine, then zidovudine/lamivudine/nevirapine since 2004; Tenofovir and efavirenz were used instead of stavudine (or zidovudine) and nevirapine for patients coinfected with hepatitis B virus. 

World Health Organization (WHO) recommends to countries two stringent methods for HIV drug resistance (HIVDR) surveillance: the HIVDR-Threshold survey to assess transmitted resistance prevalence among untreated patients and the HIVDR monitoring for patients treated during 12 months. Before constitution of an operational HIVDR task force in Niger, we conducted the present study which aimed to estimate the genetic diversity and the transmission rate of HIV-1 drug resistance in untreated patients in 2009 in Niamey, by using an alternative sampling strategy.

## 2. Materials and Methods

This prospective and analytic survey was conducted during 3 months, from October to December 2009, for plasma collection. We got samples from 96 consecutive ARV drug-naive HIV-1-infected patients, under periodic medical followup at “Centre de Traitement Ambulatoire” of Niamey, the national HIV/AIDS-Specific Care Center of Niger. 

HIV-1 RNA was extracted from plasma with the COBAS AmpliPrep Total Nucleic Acid Isolation kit (Roche), and amplified by a one-step reverse transcription-PCR using the TITAN One Tube Reverse Transcription PCR kit (Roche), with outer primers 5′P1 and 3′P1 for PR and RTAG1 and RTAG2 for RT. Nested PCR was then performed using AmpliTaq Polymerase (Applied Biosystems), with inner primers 5′P2 and 3′P2 for PR and RTAG3 and RTAG4 for RT. 

Amplified fragments, PR (codons 1–99) and RT (codons 1–240), were sequenced using the BigDye Terminator Cycle Sequencing Ready Reaction kit (Applied Biosystems), with ABI 3100 Genetic Analyzer (PE Applied Biosystems). Sequences were analysed using the Sequence Navigator software (PE Applied Biosystems), comparing the sense and antisense strands of each fragment with the prototype virus HXB2 sequence. 

For genetic subtype determination, new PR and RT sequences were aligned with sequences from reference strains of all subtypes and CRFs, using the CLUSTAL W program [4]. Phylogenetic trees were constructed using the neighbour-joining method and the Kimura two-parameter model. One hundred replicates were evaluated by phylogenetic analysis. With the bootscanning method of SimPlot software, possible recombination breakpoints and subtypes involved were determinate. 

For resistance mutation detection, amino acid sequences were compared with HXB2 consensus reference and analysed according to the 2007 International AIDS Society-United States of America (IAS-USA) and the Stanford lists of mutations [5, 6]. 

For the calculation of 95% confidence intervals (Fleiss quadratic method) and the percentages comparison, we used Epi Info 6 Software. 

This study was approved by the Ministry of Health, as a regular activity of the National Initiative for ARV Access in Niger.

## 3. Results

Out of the 96 patients, 32 were men (33%) and 64 were women (67%). The median age was 36 years (range: 23–88). All the patients have said to be heterosexual. The median of CD4 cell count was 291 cells/mm^3^ (range: 1–1145): 36.5% had more than 350 cells/mm^3^ (group 1), 34.4% had 200 to 350 cells/mm^3^ (group 2), and 29.2% had lower than 200 cells/mm^3^ (group 3). 

Phylogenetic analysis showed the presence of subtype G and five different HIV-1 Circulating Recombinant Forms (CRFs): CRF01_AE, CRF02-AG, CRF06_cpx, CRF11_cpx, and CRF30_0206. The distribution of the different genetic variant is showed in Table 1. 

After analysis of PR and RT sequences according to the 2007 IAS-USA list of mutations, 8 patients had a strain with at least one resistance mutation, so 8.3% (95% CI: 3.9–16.2%). The frequency of these strains was 8.57% (3/35), 9.09% (3/33), and 7.17% (2/28) among group 1, group 2, and group 3 patients, respectively, (*P* = 0.9611).

The different profiles are showed in Table 2. We found 4 strains with at least 1 mutation to NRTI, 5 for NNRTI and 1 for PI, so, respectively, 4.2% (CI 95%: 1.3–10.9%), 5.2% (CI 95%: 1.9–12.3%) and, 1.0% (CI 95%: 0.0–6.5%).

## 4. Discussion

For the study of HIV-transmitted (or primary) drug resistance, it is better to target recent infections (less than 6 months). It can be identified by using an indirect ELISA which quantify antibodies specific for consensus V3 peptides and consensus peptides of the immunodominant epitope gp41 [7]. This test was not done in this study, but CD4 count indicated that 63.5% of our patients had less than 350 cells/mm^3^, so already eligible for starting antiretroviral treatment, according to the recent WHO recommendations. 

The results of phylogenetic analysis confirmed the predominance of CRF02_AG in Niger, previously reported in a genetic diversity study in *env *and *gag *genes [2]. The variation of rate for this CRF, from 50% in 2000 to 56.3% in 2009, was not significant (*P* = 0.4331). CRF06_cpx decreased from 18.1% to 9.4%, but not significantly (*P* = 0.1011). The rate of CRF30_020, described for the first time in Niger, varied from 9.1% in 2000 to 15.6% in 2009 (*P* = 0.2238) [8]. Only the variation for subtype G, from 3.1% to 15.6%, was significant (*P* = 0.0031). CRF01_AE and CRF11_cpx were newly reported in the country. 

About HIVDR, these results showed that, despite the relatively recent ARV introduction in the Niger, more than 8% of untreated patients were infected by an HIV strain with at least one resistance mutation, mainly to NTRI and NNRTI. Such patients, when beginning ARV treatment, have a higher risk of virologic failure, despite the regimen originally being fully active [9, 10]. The overall prevalence of transmitted resistance was 11.5% in 2006 in Mali, a neighbouring country, using the same alternative method [11]; but studies from some others Sub-Saharan countries have reported rates lower than 5%, by using the WHO standard protocol and the WHO list of resistance mutations for epidemiological surveys [12–17]. 

According to the immunological status (CD4 number), we have not found significant difference of mutant strains rate (*P* = 0.9611).

Two CRF02_AG carried resistance mutations for two ARV classes. One strain carried Y181C and M46L. Y181CIV, as K103NS, is associated with higher levels of phenotypic resistance or clinical evidence for reduced virological response. M46L is known to be associated with tipranavir, lopinavir, and indinavir resistance. The second strain had seven mutations: M184V, L74V, M41L, L210W, T215Y, Ins69, and K103N. Two of them are not associated with resistance, but rather increase drug susceptibility: M184VI for ZDV, TDF and d4T [18, 19], and L74V for ZDV, and TDF [20, 21]. M41L, L210W and T215Y are three thymidine analog mutations (TAMs), associated with phenotypic resistance or clinical evidence for reduced virological response, mainly to stavudine and zidovudine, but also resistance to abacavir, didanosine, and tenofovir. 

In conclusion, these preliminary data confirmed the need to assure HIVDR surveillance in Niger, in order to complete the global medical monitoring of HIV patients. We plan to start in August 2011 our adapted WHO's standard protocol, including monitoring of resistance mutations for treated patients and the transmitted resistance threshold survey. These stringent conditions may offer data more comparable with those from other African countries.

## Figures and Tables

**Table 1:** Distribution of HIV-1 genetic variants among untreated patients.

Genetic variant	Number	%
Subtype G	15	15.6
CRF01_AE	1	1.0
CRF02_AG	54	56.3
CRF06_cpx	9	9.4
CRF11_cpx	2	2.1
CRF30_0206	15	15.6

Total	96	100

**Table 2:** Resistance mutations described for the 8 strains.

Patient code	Subtype	NRTI	NNRTI	PI
1799CTA09	G		K103N	
1814CTA09	G	Y115F		
1847CTA09	CRF02_AG		Y181C	M46L
1867CTA09	CRF06_cpx	T69S		
1863CTA09	CRF02_AG	T69N		
1949MVS	CRF02_AG	M41L, Ins69, L74V, M184V, L210W, T215Y	K103N	
1908CTA09	CRF02_AG		K103N	
815CTA07	CRF02_AG		K103N	
